# Risk Factors and Patterns of Drug‐Drug Interactions in Two Categories of Level‐3 Hospitals in Dhaka: A Cross‐Sectional Study

**DOI:** 10.1002/hsr2.70355

**Published:** 2025-01-14

**Authors:** Md. Abdus Samadd, Farhan Tanvir Patwary, Md Momin Islam, Ashfia Tasnim Munia, K. M. Yasif Kayes Sikdar, Md. Raihan Sarkar

**Affiliations:** ^1^ Department of Pharmacy University of Dhaka Dhaka Bangladesh; ^2^ Department of Pharmacy University of Asia Pacific Dhaka Bangladesh; ^3^ Department of Meteorology University of Dhaka Dhaka Bangladesh; ^4^ Institute of Statistical Research and Training University of Dhaka Dhaka Bangladesh; ^5^ Department of Pharmaceutical Technology University of Dhaka Dhaka Bangladesh

**Keywords:** drug‐drug interactions, pharmacodynamics, polypharmacy, prescription errors, rational use

## Abstract

**Background and Aims:**

Drug‐drug interactions (DDIs) are a significant health issue that may adversely affect the health and well‐being of patients. This study assesses and compares potential DDI (pDDI) patterns, severity, and associated risk factors in government and private hospitals in Dhaka, Bangladesh.

**Methods:**

A total of 188 and 206 prescriptions were collected from various government and private hospitals' outdoor departments, respectively, by capturing pictures of the prescriptions. Bivariate analyses were performed through STATA 15. MedScape drug interaction checker was applied to identify pDDIs, while their consequences were obtained from DrugBank and MedScape.

**Results:**

Private hospitals had more pDDIs containing prescriptions than government hospitals (62.62% and 57.97%, respectively). The mean pDDIs cases were 3.29 in the private hospitals, while at government hospitals they were 3.02. Among the detected pDDIs, pharmacodynamic pDDIs were predominat, accounting for 63.32% and 66.23% of total events in government and private hospitals, respectively. Severity‐wise, both types of hospitals had almost equal amounts of serious (10.34% vs. 9.18%), moderate (68.96% vs. 59.61%), and minor (20.06% vs. 21.79%) pDDIs. Polypharmacy was crucial in pDDI cases, responsible for 70.21% and 81.83% of pDDIs in government and private hospitals, respectively. Multiple comorbidities with pDDIs were more common in private hospitals (24.46% vs. 18.93%), while government hospitals displayed a higher frequency of pDDIs with one comorbidity (24.75% vs. 17.55%). Non‐mention of comorbidities was correlated with both types of hospitals (*p* ≤ 0.01) in pDDIs cases. Furthermore, considerable amounts of pDDIs in prescription error categories were detected. Both types of hospitals had a prevalence of antihypertensive, antidiabetic, psychotic, and antiplatelet‐related pDDIs.

**Conclusion:**

The two kinds of hospitals exhibited similar pDDI patterns, while their associations were random with the risk variables. When prescribing pharmacokinetics and pharmacodynamics pDDIs, physicians should evaluate the risk‐benefit ratio.

## 1 Introduction

A drug interaction occurs when a food, drug, or other exogenous or endogenous component alters the function of medicine; for instance, the roles of the drugs are augmented or weakened, or the combination of drugs accounts for a distinct response that none of them develops by itself [1]. When two or more drugs interact, this is described as a drug‐drug interaction (DDI). The interaction occurs when the effects of the first drug are altered by the coadministration of the subsequent drug [2]. It may be responsible for changing pharmacokinetic properties such as absorption, distribution, metabolism, excretion, and pharmacodynamic interactions such as synergism and antagonism effects or other unspecified mechanisms [3].

Drug‐drug interactions are one possible cause of adverse drug reactions (ADRs), which account for about 3%–5% of all ADRs [4]. It is one of the major sub‐types of the ADRs [5]. The Boston Collaborative Drug Surveillance Program conducted research that contained 83,200 drug intakes in about 10,000 patients and identified approximately 3600 ADRs, of which 6.5% were associated with drug interactions [6]. These adverse DDIs have been known to increase hospitalization, the period of hospital stays, morbidity, mortality, and financial costs to healthcare systems of up to $1 billion per year [7]. Annually, over 100,000 individuals die of ADRs in the United States, accounting for around 7% of all hospitalizations in Europe [1]. Geriatric patients are considered to be more vulnerable to this than some other age groups due to impairments in liver metabolism or renal performance [8]. Becker and colleagues revealed that DDIs were linked to 4.8% of hospitalizations in the aging population [5].

Some factors such as aging patients, polypharmacy, number of comorbidities, as well as the presence of prescriptions errors lead to the rise of medication errors which may responsible for amplifying the repercussion of ADRs and DDIs [5, 9, 10, 11, 12, 13]. Additionally, in 1989, Cadieux monitored that when two drugs are administered, the chance for interaction is roughly 6%; the frequency reached 50%–80% or even more while for patients taking five or more drugs concomitantly [14].

In Bangladesh, a previous study revealed that some combinations between cardiovascular drugs and other therapeutic agents such as antidiabetic, analgesic, antibiotic, and steroids related potential DDIs (pDDIs) were prominent [15]. Furthermore, another study of Dhaka city exposed that pDDIs rate was substantially higher in the comorbid patients than the non‐comorbid patients [16]. In 2021, Mamun et al. reported that the medication errors and pDDIs events were directly related to polypharmacy cases [17].

The purpose of this research was to compare the prevalence and associations of pDDIs in government and private hospitals of Dhaka, Bangladesh, based on multiple risk factors such as age, gender, polypharmacy, comorbidity, and prescription errors. Additionally, the investigation regards the frequency of severity and mechanism of the detected pDDIs. Several previous investigations have explored patterns of potential pDDIs and analyzed service quality of hospitals [15, 17, 18, 19, 20, 21]. Moreover, we recently conducted a comparative study on prescription errors and patterns in both private and government hospitals in Dhaka [22]. To the best of our knowledge, this is the first study which presents the scenario of pDDIs comparison, particularly among outpatients from private and government hospitals in Bangladesh. Although the research provides a generalized perspective, it provides a representative scenario that may be helpful for health authorities in comprehending the prevalence of pDDIs in Dhaka city.

## 2 Materials and Methods

### 2.1 Study Design

Between November 2021 and June 2022, a cross‐sectional study was performed at the outpatient departments among the randomly selected fifteen different specialized and general tertiary‐level government and private hospitals in Dhaka, Bangladesh. This research followed a similar study setting to our previous research, which focused on prescription errors and patterns [22]. Additionally, the analysis complied with the STROBE (Strengthening the Reporting of Observational Studies in Epidemiology) standards [23].

### 2.2 Inclusion Criteria of the Participants

The inclusion of the participants was in accordance with our previous study [22]. Patients of all ages who were able to answer the interview and had at least two medications prescribed were included in the study. Severe patients and pregnant women who are unable to write but can speak were included with permission from their caretaker. However, individuals who were unable to talk, except children throughout the inquiry, were excluded. For all categories of children, data were collected after taking permission of their caretaker [22].

### 2.3 Definition and Determination of Various Terms

#### 2.3.1 Determination of Polypharmacy

In this study, Polypharmacy was considered among the factors that could intensify the consequences of pDDI cases. The polypharmacy was calculated based on the Drug‐Related Problems (DRPs) literature, “prescribing drugs five or more in number” [24].

#### 2.3.2 Determination of Prescription Errors

According to Aronson (2009), “A prescription error is the failure of the prescription writing process that results in a wrong instruction about one or more of the normal features of a prescription” [25]. Among the commission and omission errors of prescriptions [26], only three different types of omission errors, such as missing age, non‐mention of comorbidities, and missing weight, were considered in this study.

#### 2.3.3 Pharmacodynamics pDDIs

While two concomitant drugs altered pharmacodynamic properties, they may provide synergistic, additive, or antagonistic effects [27].

#### 2.3.4 Pharmacokinetic pDDIs

While two concomitant drugs altered pharmacokinetic parameters of each other, such as absorption, distribution, metabolism, and excretion, they were defined as pharmacokinetic drug interactions [27].

#### 2.3.5 Unknown Mechanism

When pDDIs could not be classified under the pharmacokinetic and pharmacodynamic categories, they were defined under the unknown mechanism group.

### 2.4 Sample Size Determination

The US‐based Rasoft Inc. determined the sample size. The estimated population of Dhaka, Bangladesh, was anticipated to be over hundreds of thousands (around 230 million people) [28]. Consequently, 384 was determined to be the smallest predicted number of respondents with a degree of confidence of 95%. In addition, 3% of the population was classified as unresponsive. So, the ultimate total sample size for this study was 394.

The hospitals were chosen by the “*n* ^th^” random sampling technique. Then, the total sample size was dispersed throughout the selected hospitals by employing the proportionate distribution method, *m* = n*(*P*/*Q*). Here *m* = sample size from a particular hospital, *n* = total calculated sample size, *Q* = total bed number of the selected hospitals, *P* = bed number of a particular hospital [22, 29]. Among the randomly collected 394 prescriptions, 188 prescriptions were from government hospitals, and the rest of them (*n* = 206) were from private hospitals (Supporting Information S1: Table S1).

### 2.5 Sampling Method and Technique

A two‐step sampling strategy was used in this experiment. To collect data, many public and private hospitals' selections were considered firstly [22]. Then, participants were taken from each of the selected hospitals until the sample size was determined [22].

#### 2.5.1 Selecting Hospitals for the Investigations

Only tertiary‐level or level‐3 hospitals in Dhaka were considered for this study, while the rest were excluded. As per the Bangladeshi government database [22, 30, 31], registered tertiary‐level private and government hospitals were listed together. Hospitals' bed counts were noted using data from the hospital administration. For gathering information, the ultimate hospitals were chosen using the n^th^ sampling procedure (*n* = 2nd). Finally, 15 hospitals were selected to collect information about patients (Supporting Information S1: Table S1).

#### 2.5.2 Participants' Selections From the Selected Hospitals

In this investigation, a consecutive sampling technique [22] was employed to select respondents. All qualified individuals from the corresponding hospitals were included continually as long as the calculated sample size of selected hospitals was attained.

### 2.6 Data Collection

After completing their training, bachelor pharmacy pupils were enlisted as surveyors [22]. Data collection includes obtaining permission via a form and documenting participants' medications acquired following medical visits. Data were obtained using a one‐prescription‐per‐patient approach to ensure integrity and prevent repetition [22]. Patients' demographic information (such as age and gender), the number of prescribed medicines, occurrences of polypharmacy, the number of pDDIs, the severity and mechanism of pDDIs, and the detection of prescription errors were noted from every prescription.

### 2.7 Procedure for Potential Drug‐Drug Interactions Identification

There was no consideration for food, herbal, or mineral drug interactions. Furthermore, non‐pDDIs containing prescriptions were fully ignored for determining the factors' association between the two categories of hospitals because avoiding non‐pDDIs containing prescriptions might provide precise information of association. All potential drug‐drug interactions and their outcomes were determined using the electronic Medscape drug interaction checker and DrugBank [31, 32]. This program provided the sensitivity and specificity needed to detect pDDIs.

#### 2.7.1 Classification of Potential Drug Drug Interactions

In this study the pDDIs event was classified grounded on their severity as per the Medscape categorizations [32, 33]. Based on the severity, the pDDIs were divided into three different groups, including minor (nonsignificant), moderate, and serious (significant). Serious indicated employing a substitute, moderate signified probable deterioration of patients' clinical state and may necessitate a modification in medication, and minor (nonsignificant) indicated therapy might not need to be changed [32, 33]. Moreover, considering the pDDIs mechanism, these pDDIs were further divided into pharmacodynamic and pharmacokinetic categories. However, those pDDIs that could not be classified within the pharmacokinetic and pharmacodynamic groups were attached to the unknown mechanism section. Primarily, MedScape was employed to detect the consequences or fate of the pDDIs. Furthermore, the DrugBank database system was also utilized to add more implications to the detected pDDIs through MedScape [34, 35].

### 2.8 Statistically Analysis

All of the data were manually input into Microsoft Excel 2016 (USA). The Software for Statistics and Data Science (STATA) 15 was used to conduct the analyses (StataCorp LLC, Texas, USA). All of the pDDIs instances were input as per their therapeutic class. For comparison and representation of categorical variables such as the presence of pDDIs, types of pDDIs, polypharmacy, demographic variables (age and gender), co‐morbidities, and prescription errors, percentage and frequency were calculated. Mean value was reported for continuous variables such as number of medications and pDDIs. Additionally, the age was classified as in the previous Horng et al. study [36] because the data were collected in front of the participants, while Horng et al. divided age groups based on the facial features in their study [36]. Bivariate investigation, such as Chi‐square tests and binary logistic regression analysis were performed in this study. The Chi‐square test was used to explore the significance between the frequency of pDDIs and various factors (socio‐demographic features, prevalence of pDDIs, types of pDDIs, polypharmacy, pDDIs, number of comorbidities, and prescription errors) across the two types of hospitals. Binary regression analysis was done to evaluate the impact of hospital types, age, gender, weight, number of medicines, and prescription errors on pDDIs frequency. Among the variables, the presence of drug interactions was considered as dependent variable, while other variables were regarded as independent variables. Except for the number of medicines (continuous variables), all variables were included as categorical variables. A statistical association was considered if the *p* ≤ 0.05.

### 2.9 Data Storage, Management, Handled, and Quality Assurance

After capturing prescriptions, data were stored on a separate hard drive. Then, the raw data were gone through for critical checking to maintain the quality assurance and consistency of the data. Throughout the investigations and analysis, all data were input into Microsoft Excel version 2016. Then the data were employed for analysis. Manual data analysis and handling were totally discarded. Categorical and continuous analysis, and calculation were performed by STATA 15. To confirm data consistency and accuracy. Data collectors were instructed through an orientation class which was conducted by the corresponding author. Throughout the data‐acquiring duration, the corresponding author actively oversaw the data collection procedure, and the obtained data was reviewed daily for integrity. One week preceding the final data gathering, the collection instrument was checked on 10% of the estimated actual sample size of participants from the outdoor department of the selected hospital to ensure accuracy and consistency. The pretest data was not included in the study. The acquired data were then transmitted to a pharmacist for quality assurance before being finally analyzed.

### 2.10 Ethical Clearance

As per the similar study setting as well as the same participant inclusion criteria, the ethical permission was taken together with our previous study [22] from the “Ethical Review Committee” of the Faculty of Biological Sciences, University of Dhaka, and the ethical approval reference number was 148/Bio. Scs (attached in supplementary files). Additionally, the study adhered to the World Medical Association's (WMA) Helsinki Statement. Moreover, before receiving the participants' prescription, written or verbal consent was acquired from everyone. The consent of minors and young adults was obtained from them and their respective guardians. Privacy was strictly maintained, and the prescription information was only gathered for study. Verbal or written approval was also received directly from the hospitals' management [22].

## 3 Results

### 3.1 Sociodemographic Characteristics

The age distribution of patients differed across government and private hospitals. The percentage of adults (17–45 years old) in both sectors was comparable (35.10% vs. 38.83%, government and private, respectively). However, the frequency of older participants (> 45 years) was lower in government than in private (24.46% vs. 40.29%, respectively), leading to an overall percentage of 32.74%. The female population represented a greater proportion in both hospitals, with 62.23% in government and 58.73% in private, resulting in an overall 60.40% population (Table 1).

**Table 1:** Sociodemographic and various categorical data distribution (*n* = 394).

Topics	Government (*n* = 188) (%)	Private (*n* = 206) (%)	Total (*n* = 394) (%)
**Sociodemographic characteristics**			
**Age** (Years)			
≤ 16 (Babies and Children)	11 (5.85%)	21 (10.19%)	32 (8.12%)
17–45 (Adults)	66 (35.10%)	80 (38.83%)	146 (37.05%)
> 45 (Old adults)	46 (24.46%)	83 (40.29%)	129 (32.74%)
**Gender**			
Male	71 (37.76%)	86 (41.74%)	156 (39.59%)
Female	117 (62.23%)	120 (58.73%)	238 (60.40%)
**Medicine prescribing patterns**			
Total number of prescribed drugs	989	1,220	2,209
Average number of drugs	5.26	5.92	5.60
**Polypharmacy**			
Polypharmacy containing prescriptions	95 (50.53%)	147 (71.35%)	242 (61.42%)
pDDIs containing polypharmacy prescriptions	63 (33.51%)	91 (44.17%)	154 (39.08%)
Total pDDIs cases in polypharmacy containing prescriptions	224 (70.21%)	383 (81.83%)	607 (77.12%)
**Number of comorbidities**			
1	37 (19.68%)	88 (42.71%)	125 (31.72%)
≥ 2	77 (40.95%)	58 (28.15%)	135 (34.26%)
**Prescriptions errors**			
Absence of age	66 (35.10%)	21 (10.19%)	87 (22.08%)
Non‐mention of comorbidities	76 (40.42%)	59 (28.64%)	135 (34.26%)
Absence of weight	162 (86.17%)	184 (89.32%)	346 (87.71%)
**pDDIs prescribing patterns**			
Number of pDDIs containing prescriptions	109 (57.97%)	129 (62.62%)	238 (60.40%)
Total number of pDDIs event	330	425	755
Average number of pDDIs	3.02	3.29	3.17
**Classification of pDDIs**			
**Based on the intensity**			
Serious pDDIs in total pDDIs events	33 (10.34%)	43 (9.18%)	76 (9.65%)
Moderate pDDIs in total pDDIs events	233 (68.96%)	279 (59.61%)	512 (63.40%)
Minor pDDIs in total pDDIs events	64 (20.06%)	103 (21.79%)	167 (21.09%)
**Based on mechanism**			
Pharmacodynamics pDDIs	201 (60.90%)	301 (70.82%)	502 (66.49%)
Pharmacokinetics pDDIs	86 (26.06%)	106 (24.94%)	192 (25.43%)
Unknown mechanism	23 (6.96%)	38 (8.94%)	61 (8.07%)

### 3.2 Patterns of Prescribing Medicine and Polypharmacy

Private hospitals had a higher mean number of prescribed drugs compared to government hospitals (5.92 vs. 5.26, respectively). Polypharmacy was frequent in both government and private hospitals (50.53% vs. 71.35%, respectively). Within polypharmacy‐containing prescriptions, pDDIs appeared more frequently in private hospitals (81.83% of the total pDDIs) compared to government hospitals (70.21% of the total pDDIs) (Table 1).

### 3.3 Number of Comorbidities

Comorbidity analysis indicated that single comorbidity was higher in private hospitals than in government hospitals (42.71% and 19.68%, respectively). However, patients with two or more comorbidities were more prevalent in government hospitals than in private hospitals (40.95% and 28.15%, respectively) (Table 1).

### 3.4 Prescription Errors

A significant number of prescription errors were identified, including the absence of age (22.08%), nonmention of comorbidities (34.26%), and absence of weight (87.71%). Notably, absence of weight was more frequent in both categories of hospitals (89.32% vs. 86.17%, respectively) (Table 1).

### 3.5 Patterns of pDDIs

The results showed that 60.40% of prescriptions in the whole sample had at least one pDDI record. The average number of pDDIs in the private hospitals was a little greater than in the government hospitals (3.29 vs. 3.02, respectively) (Table 1).

### 3.6 Classification Based on Intensity

The majority were moderate pDDIs, representing 63.40% of all pDDI incidents. Serious pDDIs accounted for 9.65% of all interactions, whereas mild interactions were responsible for 21.09% (Table 1). While serious and moderate pDDIs occurred at similar frequencies in both government and private hospitals, minor pDDI cases involving prescriptions were more prevalent in government hospitals (30.09% vs. 21.27%, respectively) than private hospitals. The serious, moderate, and minor pDDIs incidents were comparable in both types of hospitals (Table S2–S7).

### 3.7 Based on Mechanism

Regarding total pDDIs across hospital types, pharmacodynamic pDDIs constituted the majority at 66.49% overall, with 60.90% occurring in government hospitals and 70.82% in private hospitals. Pharmacokinetic pDDIs followed at 25.43% overall, with 26.06% in government hospitals and 24.94% in private hospitals. Approximately 8% of pDDIs were classified as having an unknown mechanism (Table 1).

### 3.8 Factors Associated With the Occurrence of pDDIs

The binary logistic regression analysis results indicated that the number of comorbidities was linked with the incidence of pDDIs. Individuals with one comorbidity had statistically significant lower odds (OR = 0.555, CI = 0.310–0.992, *p* < 0.05) of pDDIs compared to individuals with two or more comorbidities. Though non‐mention of comorbidities was not statistically significant, the odds ratio was larger than the pDDIs event with two or more comorbidities (OR = 1.277, CI = 0.769–2.11, *p* > 0.05) (Table 2). The accuracy level of this model at the Hosmer‐Lemeshow test was 61.9%, and no significance was observed (*p* > 0.05).

**Table 2:** Binary logistic regression analysis for risk factors associated with pDDIs in various variable.

Variables	*β̂*	SE	Wald	df	*p* value	OR	95% CI for OR	
							Lower	Upper
**Hospital's type**								
Private	−0.048	0.234	0.042	1	> 0.05	0.953	0.603	1.507
**Gender**								
Female	0.132	0.218	0.366	1	> 0.05	1.141	0.744	1.751
**Age groups**								
17–45	−0.762	0.425	3.212	1	> 0.05	0.467	0.203	1.074
> 45	−0.527	0.436	1.462	1	> 0.05	0.590	0.251	1.387
**Number of medicine**	−0.068	0.068	0.996	1	> 0.05	0.934	0.818	1.068
**Presence of Polypharmacy**								
No	−0.133	0.325	0.168	1	> 0.05	0.875	0.462	1.656
**Number of comorbidities**								
1	−0.589	0.297	3.946	1	< 0.05	0.555	0.310	0.992
**Prescriptions errors**								
**Missing of age**	−0.876	0.479	3.341	1	> 0.05	0.416	0.163	1.065
**Missing of comorbidities**	0.245	0.259	0.892	1	> 0.05	1.277	0.769	2.121
**Presence of weight**								
No	−0.051	0.475	0.012	1	> 0.05	0.950	0.374	2.413
**Presence of prescription error**								
No	−0.928	0.726	1.634	1	> 0.05	0.395	0.095	1.640

*Note: p* < 0.05 was considered statistically significant.

Abbreviations: CI, confidence interval; df, degree of freedom; SE, standard errors; OR, odds ratio.

### 3.9 Associations of pDDIs Among the Government and Private Hospitals

Outcomes indicated that hospital types had no associations regarding gender and age (*p* > 0.05). However, two factors, such as moderate pDDIs and polypharmacy, exhibited lower‐level associations with the hospital categories (*p* < 0.05). Though the presence of one comorbidity showed strong significance with respect to hospital types (*p* < 0.01), two or more comorbidities had no association with the hospital types. On the contrary, the non‐mention comorbidities also demonstrated strong relationships with hospital types (*p* < 0.01) (Table 3).

**Table 3:** Various factors of pDDIs and their association among the government and private hospitals.

Topics		Government prescriptions (*n* = 188) (%)		Private prescriptions (*n* = 206) (%)		Total (*n* = 394) (%)		Chi‐square value	*p* value
		Yes	No	Yes	No	Yes	No		
**pDDIs containing prescriptions**		109 (57.97%)	79 (42.02%)	129 (62.62%)	77 (37.37%)	238 (60.40%)	156 (39.59%)	0.8858	> 0.05
**Gender**	Male	44 (23.40%)	27 (14.36%)	54 (26.21%)	31 (15.04%)	98 (24.87%)	58 (60.40%)	0.0402	> 0.05
	Female	65 (34.57%)	52 (27.65%)	75 (36.40%)	46 (22.33%)	140 (35.53%)	98 (24.87%)	1.0147	> 0.05
**Age**	≤ 16 (Babies, children)	5 (2.65%)	6 (3.19%)	10 (4.85%)	11 (5.33%)	15 (3.80%)	17 (4.31%)	0.0136	> 0.05
	17–45 (Young and Middle adults)	39 (20.74%)	27 (14.36%)	52 (25.24%)	28 (13.59%)	91 (23.09%)	55 (13.95%)	0.5378	> 0.05
	> 45 (Old adults)	23 (12.23%)	23 (12.23%)	54 (26.21%)	29 (14.07%)	77 (19.54%)	52 (13.19%)	2.789	> 0.05
**Polypharmacy**	pDDIs containing prescriptions	63 (33.51%)	46 (24.46%)	91 (44.17%)	38 (18.44%)	154 (39.08%)	84 (22.08%)	0.094	< 0.05
	Serious pDDIs containing prescriptions	21 (11.17%)	42 (22.34%)	22 (10.67%)	68 (33.00%)	43 (10.91%)	110 (27.91%)	1.4491	> 0.05
	Moderate pDDIs containing prescriptions	59 (31.38%)	4 (2.12%)	79 (38.34%)	13 (11.16%)	138 (35.02%)	27 (6.85%)	2.318	> 0.05
	Minor pDDIs containing prescriptions	26 (13.82%)	37 (19.68%)	44 (21.35%)	47 (22.81%)	70 (17.76%)	84 (21.31%)	0.753	> 0.05
**Number of comorbidities**	1	33 (17.55%)	4 (2.12%)	51 (24.75%)	37 (17.96%)	84 (21.31%)	41 (10.40%)	11.5293	< 0.01
	≥ 2	46 (24.46%)	31 (16.48%)	39 (18.93%)	19 (9.22%)	85 (21.57%)	50 (12.69%)	0.7982	> 0.05
**Various types of DDIs**	Serious pDDIs	31 (16.48%)	78 (41.48%)	33 (16.02%)	96 (46.60%)	64 (16.24%)	174 (44.16%)	0.2456	> 0.05
	Moderate pDDIs	97 (51.59%)	12 (6.38%)	102 (49.51%)	27 (13.10%)	199 (50.50%)	39 (9.89%)	4.2442	< 0.05
	Minor pDDIs	40 (21.27%)	69 (36.70%)	62 (30.09%)	67 (32.52%)	102 (25.88%)	136 (34.51%)	3.115	> 0.05
**Prescription errors**	Absence of age	43 (22.87%)	23 (12.23%)	12 (5.82%)	9 (4.36%)	55 (13.95%)	32 (8.12%)	0.439	> 0.05
	Non‐mention of comorbidities	31 (16.48%)	45 (23.93%)	38 (18.44%)	21 (10.19%)	69 (17.51%)	66 (16.75%)	7.4142	< 0.01
	Absence of weight	93 (49.46%)	69 (36.70%)	112 (54.36%)	72 (34.95%)	205 (52.03%)	141 (35.78%)	0.427	> 0.05

## 4 Discussion

Though a comparative analysis on prescription patterns and errors among the government and private hospitals in Dhaka, Bangladesh, has been completed in the same setting [22], this study is enlightening, particularly on the prevalence of pDDI patterns and comparison for the very first time. This study found a high prevalence of pDDIs in both government and private hospitals. However, no significant associations were observed with factors such as gender, older age, or polypharmacy. Interestingly, having a single comorbidity was associated with a lower number of pDDIs. Hospital type showed a statistically significant association with moderate pDDIs and polypharmacy. Notably, a stronger association was observed with the non‐mention of comorbidities and the presence of two or more comorbidities. Private hospitals had more pDDIs containing prescriptions as well as pDDI cases than government hospitals. Moderate intensity and pharmacodynamics pDDIs were more common than other pDDIs categories.

### 4.1 Patterns of pDDIs

Despite the private hospitals' prescriptions comparatively having more pDDI cases than the government hospitals' prescriptions, the outcome indicates that pDDI cases were prevalent in both types of hospitals (Table 1). They are higher than other similar studies of several South Asian countries such as Pakistan (22.3%) [37], Nepal (21.3%) [38], and Sri Lanka (52.53%) [39] but lower than an Indian study (83.25%) [40]. This discrepancy could be related to variations in survey participants, research methodology, drug prescribing and utilization trends, disease patterns, and the kinds of pDDIs evaluated. It is recommended for both types of hospitals to be careful about the occurrence of pDDIs in their prescribed drugs, especially if pDDIs were detected in more than half of total prescriptions.

### 4.2 Prevalence of pDDIs Among the Age Group Participants

A previous study accomplished in India revealed that elderly patients had a higher risk of pDDIs [40, 41] than the other age groups. Delafuente (2003) also observed that aged patients and more medicines administered led to serious drug interactions [42]. Within this study, though no correlation was found, private hospitals were more prominent here than government hospitals (26.21% vs. 17.02%, respectively). Changes in pharmacokinetics and pharmacodynamics with age raise the possibility of clinically significant drug‐drug interactions [43]. Moreover, cardiovascular, hypertensive, osteoporosis, and diabetic patients are mostly found in aged patients [44], while these kinds of pDDIs have been recorded too much in this study. So, there is a risk for the elderly patients that may influence their quality of life as morbidity increases. It is recommended to patients to take drugs as prescribed. Furthermore, prescribers in both hospitals should carefully prescribe medicines because the presence of pDDIs may adversely affect them, as elderly patients' pharmacokinetics and pharmacodynamics are vulnerable. Follow‐up is also essential to avoid medication interactions.

### 4.3 Polypharmacy and pDDIs

Polypharmacy is a significant risk factor for pDDIs. There is an association between the number of drugs administered per individual who used over four drugs and the frequency of one or more possible pDDIs [10]. The current study found that polypharmacy with pDDIs is moderately associated with the types of hospitals (*p* < 0.05). At the same time, polypharmacy‐containing prescriptions account for most of the pDDI cases in both types of hospitals. The outcomes are also similar to previous studies that occurred in the South Asian region, such as India [40], Pakistan [37], and Nepal [38], which also noticed that polypharmacy had an association with pDDI events. Doctors in Bangladesh are probably provoked or pressured to write more medicine due to excessive promotional activity from different stakeholders (pharmaceutical manufacturers, hospitals' medicine shops, etc.) [45]. However, according to the national policy, more than 70% of medicines were supplied freely from the government's budget, which may lead the prescribers of government hospitals to follow the rational use of medicines more than the private hospitals [46]. Despite the missing associations, this national policy may assist in lessening polypharmacy and pDDIs cases in government hospitals (Table 1). However, polypharmacy is practically inescapable because of the greater occurrence of comorbid problems and additional healthcare‐related issues such as using disease‐specific protocols in patient treatment and disease control [47]. So, it is recommended for both types of hospital prescribers to regard the suitability of recommended medications and the requirement of polypharmacy. Choosing nonpharmacological options or changing therapeutics where plausible are probable alternatives to avoiding polypharmacy related pDDIs.

### 4.4 Comorbidities

Comorbid conditions independently increased the pDDIs incidents by almost twofold [48]. The increasing pDDI events among hospitals with rising comorbidity cases are also detected in some previous studies, such as in India and Nepal [38, 40]. In this study, the prevalence of pDDIs was roughly similar between one or multiple comorbidities in both types of hospitals. While having one comorbidity was associated with hospital types, comparing the prevalence of pDDIs containing prescriptions between one and multiple comorbidities did not indicate which group had more pDDIs with regard to hospital types. This ambiguity might be influenced by the presence of non‐mention comorbidities or heightened physician awareness of pDDIs in cases with multiple comorbidities. Further investigation is necessary to determine which comorbidity category exhibits a higher occurrence of pDDIs.

An earlier investigation revealed that having a history of diabetes and hypertension was associated with an increased possibility of pDDIs [49]. While most frequent pDDIs in this study are recorded from the combination of comorbidities of cardiovascular diseases, hypertension, and diabetic and bacterial infections‐related diseases (Supporting Information S1: Tables S2–S7). Many ailments are treated with combination of a lot of medicines, and this strategy has a high risk of pDDIs [50]. A large number of these interactions were caused by a failure to follow treatment standards rather than by reckless professional practices [51]. Improved adherence to recommendations may reduce the incidence of such interactions [52].

### 4.5 Types of pDDIs Based on the Intensity

Among the serious pDDIs incidents, PPIs and antiplatelet (clopidogrel), the pharmacokinetic pDDI, was the most frequent pDDIs in both types of hospitals (Supporting Information S1: Table S2). This pDDI is also noted in some previous South Asian studies, such as in India [8], Nepal [38] as well as in Bangladesh [15]. Previous research found that using PPI and clopidogrel simultaneously increased the risk of mortality or hospitalization for acute coronary syndrome compared to the non‐PPI group. Moreover, histamine H‐2 receptor blockers were recommended by *Chua* et al. in place of PPIs [53]. However, the positive outcome of both hospitals is that serious pDDIs were the lowest in the perspective of other pDDIs.

On the contrary, among the moderate category pDDIs, antidiabetic, antihypertensive, and cardiovascular‐related pDDIs were the most frequently reported medications in this study (Supporting Information S1: Tables S3–S7). Such antidiabetic, antibiotic, diuretic pDDIs, as well as cardiovascular‐related pDDIs, were also observed in some previous South Asian studies, including India [54], Pakistan [37], as well as Bangladesh [15]. To reduce moderate pDDIs, rigorous monitoring and regular follow‐up over a longer period are essential for both hospitals, particularly hypertension, cardiovascular, and diabetic patients.

Minor pDDIs are more common in both types of hospitals in Dhaka city than in Pakistan (2.9%) [37], and Nepal (7.7%) [55]. Previous studies demonstrated that PPIs and vitamins are the most commonly prescribed medicines in Bangladeshi hospitals [56, 57]. Interestingly, the outcomes of this study demonstrate that the minor pDDIs belong to PPIs and vitamins in combination with diuretics, calcium supplements, CNS depressants, and antidiabetic medications (Supporting Information S1: Tables S3–S7). Though minor pDDIs are not significant for the patient's health, it is recommended for the physicians of both types of hospitals to administrate vitamins and PPIs in a balanced manner to reduce the misuse of medications. Avoiding multiple prescribing of PPIs and vitamins may effectively minimize unintended minor vitamin and PPI interactions.

### 4.6 Types of pDDIs Based on the Mechanism

Pharmacodynamics pDDIs have benefits, while jointly enhanced actions are required [58]. For example, in this study, physicians may frequently apply the combinations of antihypertensive + antihypertensive and antidiabetic + antidiabetic drugs (Supporting Information S1: Table S3–S7) to deal with chronic situations. However, pharmacodynamics pDDIs also have adverse effects, such as ondansetron and macrolides in the serious pDDIs category (Supporting Information S1: Table S2), which are responsible for QT prolongation.

The advantage of pharmacokinetic pDDIs is that they may boost medication bioavailability and maintain drug plasma concentrations inside the therapeutic range for longer. They may be performed by increased barrier permeation, transport molecule modulation, and metabolic enzyme suppression [40]. However, increasing or decreasing drug concentration through pharmacokinetic interactions may affect patients' safety and therapeutic effectiveness.

While both of the hospitals contained more than 20% of pharmacokinetic pDDIs and 60% of pharmacodynamic pDDIs, and they both have safety issues, both types of hospitals need to check up pharmacokinetic pDDIs; are they safe or not? It is especially for elderly, pregnant, hypertensive, cardiovascular, diabetic, and psychotic disorder patients.

### 4.7 pDDIs and Prescription Errors

Comorbidities, age, and weight are needed to assess the risk‐benefit ratio. Except for missing age, both hospitals have roughly the same number of absence of weight cases. The consequences of drug‐drug interactions vary from age to age, especially for geriatrics, because of the alteration of the pharmacokinetic mechanism of drugs [59]. So, missing age is a hazard to calculating the risk‐benefit ratio, whether or not a patient can tolerate the adverse effects of pDDIs. Drug distribution, metabolism, and clearance are vastly affected by the obesity of the patients [60]. Without weight, it is difficult to calculate the risk‐benefit ratio of the pDDIs, especially pharmacokinetics pDDIs, because they vastly depend on the weight (obesity) of the patients. Conversely, private hospitals showed a slightly higher incidence of non‐mention comorbidities and pDDIs compared to government hospitals (Table 3). The higher prevalence of polypharmacy in private hospitals might explain the increased occurrence of pDDIs. Moreover, the reasons for the higher incidence of non‐mention comorbidities in private hospitals compared to government hospitals are not entirely clear; stringent regulatory oversight in government hospitals could potentially play a role in this difference. Comorbidities are needed to assess the benefits and hazards of the pharmacodynamics and pharmacokinetic of pDDIs.

During the prescribing, it is crucial to detect pDDIs to minimize the unwanted consequences. However, it is challenging to care for every pDDI at the time of prescribing several concomitant drugs, especially in polypharmacy cases [52]. Electric prescriptions with the facility of the pDDIs detecting software may be beneficial to detect and reduce unwanted pDDIs during prescribing. Internet‐based software, as well as digitalization of the health care system, makes a considered amount of deduction (66%) of pDDIs at the time of prescribing [61].

Though preventing the 100% occurrence of pDDIs may not be achievable, continuous monitoring, as well as pDDIs surveillance, can reduce the life‐threatening consequences. These can be done by healthcare professionals and patients at the very beginning by following some guidelines, such as using detection software by professionals and maintaining the accuracy of drug intake by patients. It is recommended to seek alternate medications with fewer interactions if possible and use algorithms to analyze the degree of probable interactions. As a result, continuous monitoring and management of patients is required for better treatment results.

## 5 Limitation of the Study

Several things could be improved in this research. Only the outdoor department was considered, while the indoor department was entirely ignored. Furthermore, pDDI events were screened from the Medscape and DrugBank; other sources of pDDIs screening were totally ignored. Only medications were counted, but their dosages, administration routes, and frequency were strictly avoided. Additionally, the information was gathered during the COVID‐19 outbreak, which presents a health concern.

## 6 Conclusion and Recommendations

The findings provide tentative information on the extent of pDDIs in outdoor departments at government and private hospitals in Dhaka, Bangladesh. The outcomes illustrated that pDDIs pattern and associations are random, and both private and government hospitals in Dhaka city are vulnerable to pDDIs. Among the risk factors, pDDIs events were only associated with the number of comorbidities. Both hospitals; pDDIs patterns is identical to those of the other South Asian countries, as well as contain a huge number of pDDI incidents. The serious pDDIs are the same in both types of hospitals. In contrast, private hospitals' prescriptions have more minor pDDIs, and government hospitals' prescriptions are far up in moderate pDDIs. There is some recommendation for both types of hospitals to maintain pDDIs events.

- Careful medication use and monitoring are required to reudce drug‐drug interaction.
- Calculate the risk‐benefit ratio to recommend pharmacodynamic and pharmacokinetic pDDIs to geriatric, hypertensive, osteoporotic, and diabetic patients.
- Lowering polypharmacy, seeking nonpharmacological options, frequently re‐evaluating treatment options, adjusting dosing regimens, and changing dosages may reduce pDDIs in both hospitals.
- Because of the lack of electronic prescriptions in both types of hospitals, it is highly recommended to use software‐assisted electronic prescriptions, which can automatically check during the prescribing of drugs and decide whether pDDIs are safe for patients.
- Both hospitals should implement pDDIs surveillance, patient monitoring education and counseling, and routine follow‐up for outdoor patients.

## Author Contributions

**Md. Abdus Samadd:** conceptualization, formal analysis, investigation, methodology, software, visualization, writing–original draft. **Farhan Tanvir Patwary:** data curation, formal analysis, investigation, methodology, software, writing–original draft. **Md Momin Islam:** conceptualization, formal analysis, investigation, methodology, writing–original draft. **Ashfia Tasnim Munia:** investigation, methodology, writing–original draft. **K. M. Yasif Kayes Sikdar:** conceptualization, methodology, writing–original draft, writing–review and editing; supervision. **Md. Raihan Sarkar:** methodology, writing–original draft, writing–review and editing, supervision.

## Conflicts of Interest

The authors declare no conflicts of interest.

## Transparency Statement

The lead author K. M. Yasif Kayes Sikdar, Md. Raihan Sarkar affirms that this manuscript is an honest, accurate, and transparent account of the study being reported; that no important aspects of the study have been omitted; and that any discrepancies from the study as planned (and, if relevant, registered) have been explained.

## Supporting information

Supporting information.

## Data Availability

The data that support the findings of this study are available on request from the corresponding author. The data are not publicly available due to privacy or ethical restrictions.
