# Supplementary material for: Risk Factors and Patterns of Drug‐Drug Interactions in Two Categories of Level‐3 Hospitals in Dhaka: A Cross‐Sectional Study
Source: Health Sci Rep. 2025 Jan 14;8(1):e70355. doi: 10.1002/hsr2.70355 (PMC11730072; doi:10.1002/hsr2.70355)
Supplement: Supplementary file 1 — Supporting information. [file HSR2-8-e70355-s001.docx]

**Risk Factors and Patterns of Drug‐Drug Interactions in Two Categories of Level‐3 Hospitals in Dhaka: A Cross‐Sectional Study**

Md Abdus Samadd^1^, Farhan Tanvir Patwary^2^, Md. Momin Islam^3^, Ashfia Tasnim Munia^4^, K. M. Yasif Kayes Sikdar^5*^ and Md. Raihan Sarkar^5^

^1^ Department of Pharmacy, University of Dhaka, Dhaka, Bangladesh.

^2^ Department of Pharmacy, University of Asia Pacific, Dhaka, Bangladesh.

^3^ Department of Meteorology, University of Dhaka, Dhaka-1000, Bangladesh

^4^ Institute of Statistical Research and Training, University of Dhaka, Dhaka, Bangladesh.

^5^ Department of Pharmaceutical Technology, University of Dhaka, Dhaka, Bangladesh.

**Email addresses:**

**Md Abdus Samadd:** [abdus-2015018058@pharmacy.du.ac.bd](mailto:abdus-2015018058@pharmacy.du.ac.bd)

**Farhan Tanvir Patwary:** [tanvirfarhan@outlook.com](mailto:tanvirfarhan@outlook.com)

**Md. Momin Islam:** [momin@du.ac.bd](mailto:momin@du.ac.bd)

**Ashfia Tasnim Munia:** [atasnim@isrt.ac.bd](mailto:atasnim@isrt.ac.bd)

**K. M. Yasif Kayes Sikdar:** [yasif@du.ac.bd](mailto:yasif@du.ac.bd)

**Md. Raihan Sarkar:** [raihan.rezvi@du.ac.bd](mailto:raihan.rezvi@du.ac.bd)

***Correspondence to:**

K. M. Yasif Kayes Sikdar and Md. Raihan Sarkar

Email: [yasif@du.ac.bd](mailto:yasif@du.ac.bd) and [raihan.rezvi@du.ac.bd](mailto:raihan.rezvi@du.ac.bd)

**Table of contents**

**Table S1:** Selected hospitals for the prescription collection through the “nth simple random sampling technique”.

**Table S2:** Serious pDDIs combinations of both types of hospitals

**Table S3:** Topmost antidiabetic containing moderate and minor pDDIs combinations of both types of hospitals

**Table S4:** Topmost antihypertensive containing moderate and minor pDDIs combinations of both types of hospitals

**Table S5:** Topmost central nervous system (CNS) depressant containing moderate and minor pDDIs combinations of both types of hospitals

**Table S6:** Topmost antiplatelet containing moderate and minor pDDIs combinations of both types of hospitals

**Table S7:** Topmost NSAIDs containing moderate and minor pDDIs combinations of both types of hospitals

**Table S1**: Selected hospitals for the prescription collection through the “nth simple random sampling technique”.

| **Government hospitals** | **Frequency**  **(percentage)** | **Private hospitals** | **Frequency**  **(percentage)** |
| --- | --- | --- | --- |
| National Heart Foundation Hospital and Research Institute | 25  (13.30%) | Islami Bank Central Hospital | 19  (9.22%) |
| Dhaka Medical College Hospital | 38  (20.21%) | BIRDEM General Hospital | 32  (15.53%) |
| National Institute of Cardiovascular Diseases | 19  (10.11%) | Anwar Khan Modern Medical College Hospital | 24  (11.65%) |
| Shaheed Suhrawardy Medical College and Hospital | 26  (13.83%) | LABAID Specialized Hospital | 19  (9.22%) |
| Mugda Medical College and Hospital | 18  (9.57%) | Evercare Hospital Dhaka | 26  (12.62%) |
| Sir Salimullah Medical College Hospital | 28  (14.89%) | Ibn Sina Specialized Hospital | 25  (12.14%) |
| Bangabandhu Sheikh Mujib Medical University Hospital (BSMMU) | 34  (18.09%) | Square Hospital Private Limited | 33  (16.02%) |
|  |  | Green Life Medical College Hospital | 28  (13.59%) |
| Total | 188  (100.00%) |  | 206  (100%) |

Seven different government hospitals and eight different private hospitals were selected through the “nth” random sampling technique at the time of the data collection (Table S1).

**Table S2:** Serious pDDIs combinations of both types of hospitals:

| **Interacted drugs** | **Clinical outcome** | **Government DDIs (n = 319) (%)** | **Private DDIs (n = 468) (%)** |
| --- | --- | --- | --- |
| Antiplatelet + PPIs | PPIs decreases antiplatelet’s (clopidogrel) efficacy via suppressing CYP2C19. | 19 (5.95%) | 22 (4.70%) |
| Tri-cyclic antidepressant (TCA) + TCA | The metabolism of TCA such as amitriptyline can be decreased and increase serotonin levels when combined with TCA (duloxetine). | - | 4 (0.85%) |
| Antiplatelet + Antihypertensive | Pharmacodynamic antagonism: co-administration reduces renal function. Antiplatelet aspirin inhibits the production of vasodilating renal prostaglandins. | - | 3 (0.64%) |
| Cephalosporin antibiotic + Anticoagulant | Cephalosporin antibiotic (ceftriaxone) promotes anticoagulant’s (enoxaparin) activity. | 2 (0.62%) | **-** |
| Antifungal + Antifungal | Fluconazole with ketoconazole prolongs QTc (corrected for heart rate) by decreasing metabolism of ketoconazole. | 2 (0.62%) | **-** |
| Antilipemic agents + HMG-CoA reductase inhibitors | Pharmacodynamic synergism. Fenofibrate can increase rhabdomyolysis probability while combined with a statin treatment to reduce triglyceride and enhance high-density lipoproteins. | 4 (1.25%) | 4 (0.85%) |
| Macrolide antibiotic + 5-hydroxytryptamine (5-HT3)  Antagonist | 5-HT3 antagonist (Ondansetron) with Macrolide antibiotic (azithromycin) raises QTc. | 1 (0.31%) | 4 (0.85%) |
| Non-steroidal anti-inflammatory drug (NSAID) + NSAID | Pharmacodynamic synergy amplifies toxicity. | 2  (0.62%) | 1 (0.21%) |
| Antifungal+ PPI | By elevating stomach pH, PPI (esomeprazole) diminishes antifungal’s (ketoconazole) potency by reducing absorption. | 4 (1.25) | 3 (0.64%) |
| **Total** |  | 33 (10.34%) | 43 (9.18%) |

The pDDI between antiplatelet (clopidogrel) and proton pump inhibitors (PPIs) was the most widely prescribed serious pDDI combination in both kinds of hospitals (5.95% government vs. 4.7% private) (Table S2). PPIs suppress the CYP2C19 enzyme required to activate clopidogrel, leading to pharmacological incompetency. Antilipemic agents (fenobrate) in combination with HMG-CoA reductase inhibitors as well as ketoconazole in combination with PPIs were two others frequently found serious pDDIs in both types of hospitals (Table S2).

**Table S3:** Topmost antidiabetic containing moderate and minor pDDIs combinations of both types of hospitals:

| **Types of pDDIs** | **Interacted drugs** | **Clinical outcomes** | **Government DDIs (n = 319) (%)** | **Private DDIs (n = 468) (%)** |
| --- | --- | --- | --- | --- |
| **Moderate pDDIs** | Antidiabetic | Each boosts the other's effect through pharmacodynamic synergy may cause hypoglycemia. | 37 (11.59%) | 52 (11.11%) |
|  | Antihypertensive | Angiotensin II receptor blockers such as losartan and olmesartan boost insulin aspart effects in an unknown way, while Angiotensin-converting-enzyme (ACE) inhibitor (ramipril) increases metformin toxicity via pharmacodynamically interaction | 26 (8.15%) | 27 (5.76%) |
|  | Calcitriol | Calcitriol increases antidiabetic drugs such as linagliptin effects by affecting CYP3A4 metabolism. | 4 (1.25%) | 8 (1.70%) |
| **Minor pDDIs** | vitamin B12 | Antidiabetic (metformin) reduces vitamin B12 in an unknown manner and can cause vitamin B12 deficiency after several years. | 8 (2.50%) | 17 (3.63%) |
|  | Diuretics | Antidiabetic (metformin) reduces diuretic activity (furosemide) in an unknown manner. | 2 (0.62%) | 8 (1.70%) |
|  | Testosterone | Pharmacodynamic synergism. | 2 (0.62%) | - |

**Table S4:** Topmost antihypertensive containing moderate and minor pDDIs combinations of both types of hospitals:

| **Types of DDIs** | **Interacted drugs** | **Clinical outcomes** | **Government DDIs (n = 319) (%)** | **Private DDIs (n = 468) (%)** |
| --- | --- | --- | --- | --- |
| **Moderate pDDIs** | Antihypertensive | Pharmacodynamic pDDIs. | 31 (9.71%) | 37 (7.90%) |
|  | Antidiabetic | Angiotensin II receptor blockers such as losartan and olmesartan boost insulin aspart effects in an unknown way, while ACE inhibitor (ramipril) increases metformin toxicity via pharmacodynamically interaction. | 26 (8.15%) | 27 (5.76%) |
|  | Antiplatelet | Antiplatelet (aspirin) interacts with antihypertensive drugs angiotensin II receptor blocker (valsartan, losartan), and beta-blocker (metoprolol, salmeterol, carvedilol) in pharmacodynamically manner. | 23 (7.21%) | 17 (3.63%) |
|  | Calcium Carbonate & Sodium Bicarbonate | Calcium carbonate blocks antihypertensive effects such as calcium channel blockers (amlodipine) in a pharmacodynamic manner. Furthermore, calcium carbonate and sodium bicarbonate pharmacokinetically inhibit gastrointestinal (GI) absorption in beta-blockers (labetalol, bisoprolol). | 17 (5.32%) | 28 (5.97%) |
|  | NSAID | In combination, NSAID reduces the antihypertensive activity of angiotensin II receptor blockers (olmesartan, losartan) and beta-blocker (propranolol). | 5 (1.56%) | 13 (2.77%) |
|  | SSRI | An antipsychotic agent such as SSRI (sertraline) increases beta-blocker (propranolol) levels by affecting CYP2D6 metabolism in a pharmacokinetic manner. | 4 (1.25%) | 3 (0.64%) |
|  | Diuretics | Diuretics (spironolactone, furosemide) may increase the excretion rate of beta-blocker (bisoprolol), which could result in a lower serum level and potentially a reduction in efficacy in pharmacokinetics way. The combination of both agents increases serum potassium levels in an unknown manner. | 9 (2.82%) | 9 (1.92%) |
| **Minor pDDIs** | SSRI | SSRI (escitalopram) slows the metabolism of antihypertensive beta-blocker (bisoprolol) amounts. | - | 3 (0.64%) |

**Table S5:** Topmost central nervous system (CNS) depressant containing moderate and minor pDDIs combinations of both types of hospitals:

| **Types of pDDIs** | **Interacted drugs** | **Clinical outcomes** | **Government DDIs (n = 319) (%)** | **Private DDIs (n = 468) (%)** |
| --- | --- | --- | --- | --- |
| **Moderate pDDIs** | Antihistamine | Benzodiazepine (clonazepam) and antihistamine (cinnarizine, dimenhydrinate) both cause of sedation. | 2 (0.62%) | 8 (1.70%) |
|  | Antipsychotic | Benzodiazepine drugs (alprazolam) and antipsychotic (quetiapine) combination may be responsible for sedation. | 5 (1.56%) | 5 (1.06%) |
|  | Pregabalin | Each boosts the other's effect through pharmacodynamic synergy. | 1 (0.31%) | 4 (0.85%) |
|  | gamma-aminobutyric acid (GABA) receptor agonist | Benzodiazepine (clonazepam) with the combination of GABA receptor agonist (Baclofen) induce sedation. | 1 (0.31%) | 4 (0.85%) |
| **Minor pDDIs** | PPIs | PPIs (omeprazole, esomeprazole) boosts amounts of benzodiazepine (clonazepam) via altering liver enzyme CYP2C19 metabolism | 29 (9.09%) | 15 (3.20%) |
|  | Vitamin B12 | CNS depressant benzodiazepine (clonazepam) limits GI absorption, which reduces vitamin B12 levels. | - | 12 (2.56%) |
|  | NSAID | CNS depressant benzodiazepine (clonazepam) boosts NSAID (acetaminophen) metabolism. | 7 (2.19%) | 4 (0.85%) |
|  | Fluoroquinolone antibiotics | By reducing metabolism, fluoroquinolone antibiotics (moxifloxacin) promote benzodiazepine (clonazepam) amounts. | 3 (0.94%) | - |
|  | PPIs | Esomeprazole increases chlordiazepoxide by delaying metabolism. | 3 (0.94%) | - |

**Table S6:** Topmost antiplatelet containing moderate and minor pDDIs combinations of both types of hospitals:

| **Types of DDIs** | **Interacted drugs** | **Clinical outcome** | **Government DDIs (n = 319) (%)** | **Private DDIs (n = 468) (%)** |
| --- | --- | --- | --- | --- |
| **Moderate pDDIs** | Antihypertensive | Antiplatelet such as aspirin interacts with antihypertensive agent, including angiotensin II receptor blocker (valsartan, losartan) and beta-blocker (metoprolol, salmeterol, carvedilol) in a pharmacodynamically manner | 23 (7.21%) | 17 (3.63%) |
|  | Anticoagulant | Each boosts the other's toxicity through pharmacodynamic synergy. | 4 (1.25%) | 6 (1.28%) |
|  | Vasodilator | Aspirin increases nitroglycerin's effects by vasodilation. | 11 (3.44%) | 10 (2.13%) |
|  | Antiplatelet | Each boosts the other's toxicity through pharmacodynamic synergy. | 6 (1.88%) | 4 (0.85%) |
|  | Diuretics | Aspirin reduces diuretic (spironolactone, frusemide) effects in an unknown way. | 5 (1.56%) | - |
|  | PPIs | Pantoprazole decreases clopidogrel effects by affecting CYP2C19 metabolism. | 3 (0.94%) | - |

**Table S7:** Topmost NSAIDs containing moderate and minor pDDIs combinations of both types of hospitals:

| **Types of DDIs** | **Interacted drugs** | **Clinical outcome** | **Government DDIs (n = 319) (%)** | **Private DDIs (n = 468) (%)** |
| --- | --- | --- | --- | --- |
| **Moderate pDDIs** | Antihypertensive | In combination, NSAID reduces the antihypertensive activity of angiotensin II receptor blockers (olmesartan, losartan) and beta-blocker (propranolol) | 5 (1.56%) | 13 (2.77%) |
| **Minor pDDIs** | Vitamin B12 | NSAID reduces vitamin B12 via decreasing GI absorption. | - | 2 (0.42%) |
|  | CNS depressant | CNS depressant benzodiazepine (clonazepam) boosts NSAID (acetaminophen) metabolism. | 7 (2.19%) | 4 (0.85%) |
|  | Calcium Carbonate | Passive renal tubular reabsorption owing to higher pH | 2 (0.62%) | 2 (0.42%) |
|  | Vitamin C | Vitamin C (ascorbic acid) increases NSAIDs (aspirin) amount via competing for renal tubular clearance. | - | 4 (0.85%) |

**Moderate pDDIs**

Both types of hospitals featured nearly the same kinds of moderate pDDIs. For example, Antidiabetic + Antidiabetic, Antihypertensive + Antihypertensive, Antihypertensive + Antidiabetic, Antiplatelet + Antihypertensive, and Calcium Carbonate + Antihypertensive such pDDIs were the most frequent and common pDDIs in both types of hospitals' prescriptions (Table 4-8). However, the topmost frequent moderate pDDIs in both hospitals were cardiovascular and diabetes related (Table S3-S7). In both types hospitals, Antidiabetic + Antidiabetic combinations were the topmost moderate pDDIs, (11.59% of the government's total vs. 11.11% of private's total) (Table S3-S7). However, the topmost frequent moderate pDDIs in both hospitals were cardiovascular and diabetes-related pDDIs (Table S3-S7).

Calcium channel blocker (amlodipine) acts as a pharmacodynamic antagonist of antidiabetic (metformin), whereas two antidiabetic combinations, linagliptin and insulin, exert a pharmacodynamic synergistic effect. Various mental disorder-related pDDIs, such as the combination of SSRIs (sertraline) and anti-psychotic (mirtazapine), were also recorded in the moderate pDDI result. The sertraline with mirtazapine combination boosts serotonin secretion. This study also detected pDDIs between diuretics, including spironolactone and furosemide and other medicines like antihypertensive and antibiotics.

**Minor pDDIs**

PPIs, CNS depressants, antidiabetic, and Vitamin B12 drugs and their combinations were the most often found in minor pDDIs in both types of hospitals (Table S3-S7). PPIs + CNS depressant combination was the most frequent pDDIs in government prescriptions (9.09% in total), while private prescriptions had PPIs + Vitamin B12 (5.98% in total) pair in the most frequent pDDIs (Table 4-8).In both types of hospitals antidiabetic + antidiabetic combinations were the topmost moderate pDDIs, (11.59% of government’s total vs 11.11% of private’s total) (Table S3-S7).

In this instance of minor pDDIs interactions, vitamins B12 and B9 were more dominant than other vitamins. Both the CNS depressant benzodiazepine (clonazepam) and the PPIs (dexlansoprazole, omeprazole, and esomeprazole) interact pharmacokinetic way to reduce vitamin B12 absorption. Another common minor pDDI combination PPIs and CNS depressants (clonazepam); PPIs increase the amount of clonazepam through the change in metabolism.

**Pharmacodynamics pDDIs**

Physicians occasionally apply pharmacodynamics synergism pDDIs to treat chronic illnesses, such as the administration of insulin in conjunction with linagliptin or Metformin for treating chronic diabetes, whereas beta-blocker (bisoprolol) and angiotensin II receptor antagonists (losartan) are used in combination to control hypertension (Table S3-S7). However, physicians should be aware of the possible adverse effects of combination uses. For instances, *Gupta et al*. noticed in their study that there was a possible risk of adverse effects such as postural hypertension, among patients who received two antihypertensive agents in the beginning [1]. In the future, controlled trials will be needed to determine if correct clinical treatment of pDDIs can reduce illness and death caused by medications.

**References**

1. Gupta AK, Arshad S, Poulter NR. Compliance, Safety, and Effectiveness of Fixed-Dose Combinations of Antihypertensive Agents. Hypertension. 2010;55: 399–407. doi:10.1161/hypertensionaha.109.139816
